# Characteristics and Degradation Mechanisms under High Reverse Base–Collector Bias Stress in InGaAs/InP Double HBTs

**DOI:** 10.3390/mi14112073

**Published:** 2023-11-08

**Authors:** Silu Yan, Hongliang Lu, Lin Cheng, Jiantao Qiao, Wei Cheng, Yuming Zhang

**Affiliations:** 1 Key Laboratory for Wide Band Gap Semiconductor Materials and Devices of Education Ministry, School of Microelectronics, Xidian University, Xi’an 710071, China; siluyan_xidian@163.com (S.Y.); domini_c@foxmail.com (L.C.); 22111213679@stu.xidian.edu.cn (J.Q.); zhangym@xidian.edu.cn (Y.Z.); 2 Science and Technology on Monolithic Integrated Circuits and Modules Laboratory, Nanjing Electronic Devices Institute, Nanjing 210016, China; dspbuilder@163.com

**Keywords:** heterostructure bipolar transistor (HBT), indium phosphide, TCAD modeling, reliability, electrical stress

## Abstract

In this paper, the reliability of InP/InGaAs DHBTs under high reverse base–collector bias stress is analyzed by experiments and simulation. The DC characteristics and S parameters of the devices under different stress times were measured, and the key parameters with high field stress were also extracted to fully understand and analyze the high-field degradation mechanism of devices. The measurements indicate that the high-field stress leads to an increase in base current, an increase in base–collector (B–C) and base–emitter (B–E) junction leakage current, and a decrease in current gain, and different degrees of degradation of key parameters over stress time. The analysis reveals that the degradation caused by reverse high-field stress mainly occurs in the B–C junction, access resistance degradation, and passivation layer. The physical origins of these failure mechanisms have been studied based on TCAD simulation, and a physical model is proposed to explain the experimental results.

## 1. Introduction

InP-based HBTs have been widely used in ultra-high-speed digital-to-analog hybrid circuits [1,2,3] and the mm and sub mm wave circuit designs [4,5,6] mainly due to the superior characteristics of high-frequency gain, high power density, low white noise, good linearity and so on. With the continuous improvement of application requirements, devices are continuously scaling down in size to increase frequency characteristics and meet the corresponding circuit speed. However, that also makes the peak operating current density higher and higher, which not only leads to a more serious self-heating effect but also makes the electric field of the device in the collector junction more concentrated (at a fixed junction voltage) and the breakdown voltage tends to decrease, resulting in an impact on the device reliability which cannot be ignored. Therefore, an adequate and fine InP HBT failure analysis is not only helpful in proposing effective improvements in device reliability, but can also be integrated its compact model to predict the circuit’s long-term operation, which is of great significance to ensure the reliability of circuit and system performance.

Several research institutes have studied the reliability of HBTs under mixed-mode stress and reverse bias stress through experiments [7,8] and TCAD modeling [8,9]. Several researchers have reported the reliability studies of InP-based HBTs. For example, G.A. Kone et al. carried out accelerated aging tests under thermal and electrical stresses on InGaAs/InP DHBTs of a 0.7 μm process [10,11] by experiments and TCAD simulation. The results indicate that the device failure under electro-thermal stress is mainly due to the traps introduced at the emitter–sidewall/passivation interface, the B–E junction, and the increase in the emitter resistance. Y. K. Fukai et al. studied the reliability of sub-micrometer, high-speed and low-power InP HBTs with a 0.6 × 3 μm^2^ emitter size at high current densities [12], and pointed out that the degradation of the B–E junction is the main factor leading to the failure of devices under high current densities. Hong Wang et al. reported the reliability of InGaAs/InP DHBTs with two different emitters areas (emitter area of 5 × 20 μm^2^ and 40 × 40 μm^2^) under a high reverse B–C bias voltage (avalanche) regime [13]. The experimental studies suggest that the degradation of the device is caused by the increase in the generation–recombination centers localized at B–E and B–C junction peripheries caused by hot carriers which are generated in the reverse-bias B–C junction due to the impact ionization. The decrease in the current gain caused by the stress is more obvious in the device with a smaller size. Later, they also carried out research on the characteristics of RF and noise characteristics of InP/InGaAs DHBTs with an emitter area of 1.6 × 20 μm^2^ [14]. The result reflects that the degradation of RF performance is more significant than that of DC performance by comparing the performance before and after stress.

For a DHBT during microwave power operation, the base–collector junction is usually highly reverse-biased [13], and to meet the requirements of current applications, the device would also be forced to operate near the safe operating area (SOA) [8], some of which leads the device to high reverse-biased conditions. Under such operating conditions, the device’s reliability will be affected, reducing the circuit’s lifetime in the long run. However, it can be found from the previous reports that the studies on the reliability of HBTs are focused on the stress without avalanche multiplication, in which situation the reverse bias applied on the collector junction is usually conservative. Although Hong Wang et al. have relatively comprehensively studied and analyzed the reliability of DC characteristics of devices under high-reverse-bias conditions, they mainly focus on large-sized devices. However, the scaling down of devices’ size will make the electric field intensity in the collector area increase continuously (under the fixed junction voltage), and various edge effects become more prominent. Thus, it is more relevant to understanding the device degradation under high B–C junction reverse bias conditions. Moreover, the radio frequency (RF) characteristics, as an important indicator of devices’ performance, also require a comprehensive analysis under the high-junction-reverse-bias condition to completely assess and improve the high-field performance and reliability of equipment, which is seldomly analyzed in the degradation process in key parameters with stress time in previous studies.

In this paper, the degradation of 0.7 × 10 μm^2^ InP/InGaAs/InP DHBTs under high B–C reverse bias stress has been reported. A detailed study of the degradation behavior of the DC characteristics and key parameters under high B–C reverse bias stress is presented. A physical model is also proposed to explain the device degradation mechanism. This paper is organized as follows. In Section 2, the InGaAs/InP DHBT technologies and the stress conditions are presented. In Section 3, the test results are described and discussed. The device failure mechanisms performed at different stages during the tests are analyzed using TCAD simulations in Section 4.

## 2. Description of Devices and Tests

### 2.1. Device Description and Experimental Conditions

**Figure 1:**
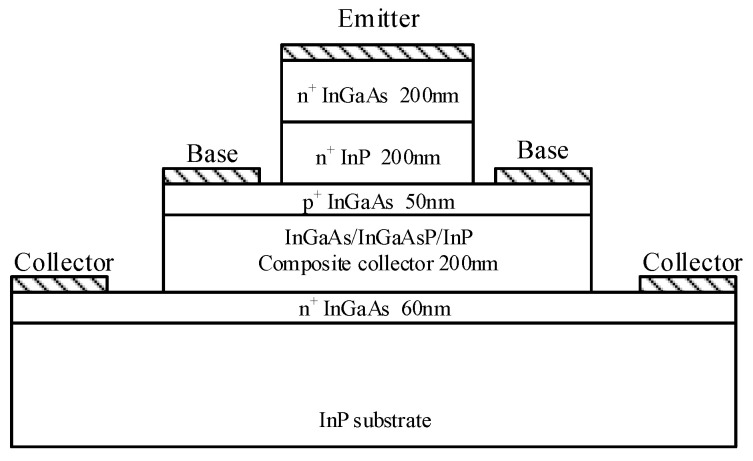
Layer structure of the InGaAs/InP DHBT.

**Figure 2:**
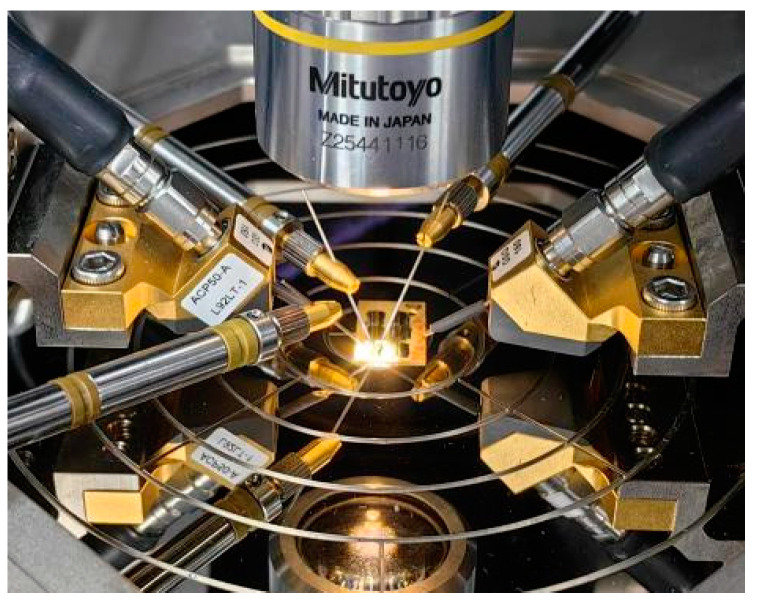
On-wafer measurement setup.

The devices used in this paper adopted a three mesa-structure. The structure diagram is shown in Figure 1, in which the Emitter, Base and Collector are, respectively, labeled. The DHBT structure includes an InGaAs cap layer (200 nm, 3 × 10^19^ cm^−3^), an InP emitter (200 nm, 2 × 10^17^ cm^−3^), a carbon-doped InGaAs base (50 nm, 3 × 10^19^ cm^−3^), and a compositionally step-graded InGaAs/InGaAsP/InP collector (200 nm, 1 × 10^16^ cm^−3^). Non-alloy Ti/Pt/Au and Pt/Ti/Pt/Au metallization have been used for N-type and P-type ohmic contacts, respectively. The device with a 0.7 × 10 μm^2^ emitter was chosen for the stress experiments. The stress experiments were conducted at room temperature using a Keysight B1500A Semiconductor Parameter Analyzer. To accurately evaluate the influence of high-reverse-bias electrical stress on device characteristics, on-wafer S-parameter measurements were employed at preselected stress times to obtain the S-parameter over the frequency range from 100 MHz to 40 GHz using a Rohde & Schwarz ZVA 50 Vector Network Analyzer (10 M–50 GHz) and Keysight B2902A controlled by Keysight’s Integrated Circuit Characterization and Analysis Program (IC-CAP)), which is used for extracting the key parameters of the device. Moreover, the on-wafer calibration was carried out before the electrical stress measurement. In addition, more attention should be paid to maintaining the stability of the test environment (ambient temperature: 25 ± 2 °C, humidity: 47 ± 5%) to ensure the calibration conditions would not deviate during the experiments. Both the DC probe and the RF probe are installed on the RF probe table. After the electrical stress, the DC probe is quickly replaced with the RF probe to avoid any test error caused by the time of replacing the probe. The test equipment is shown in Figure 2.

### 2.2. Stress Conditions and Experiments

Before the experiment, it is necessary to determine the high-bias stress conditions first. The breakdown voltage of the B–C junction (BV_CBO_) of the device is 4.7 V. Therefore, the reverse high-field voltage applied on the B–C junction is chosen as 4 V by experiments, which is about 85% of BV_CBO_ [14]. The selected reverse high-field voltage is lower than the B–C junction breakdown voltage (4.7 V) but higher than the nominal operating voltage of the device which is usually less than 2 V. This value is chosen as a reasonable trade-off between keeping stress conditions close to the operating ones and obtaining a non-negligible degradation in a reasonable amount of time.

During stress, a constant B–C reverse bias (V_BC_ = −4 V) was applied to the collector while holding the emitter open. The B–C leakage current was measured at V_BC_ = −2 V every 60 s, which was adopted to monitor the degradation of the B–C junction during the stress. And the B–E and B–C junction characteristics and gummel curves of the device were tested every 300 s during stress. The stress conditions were interrupted at pre-selected times so that the S-parameter of the devices could be measured to extract the key parameters. All measurements were implemented at room temperature.

## 3. Effect of Stress on Device Characteristics

### 3.1. Stress-Induced Leakage Current in the B–C Junction and B–E Junction

**Figure 3:**
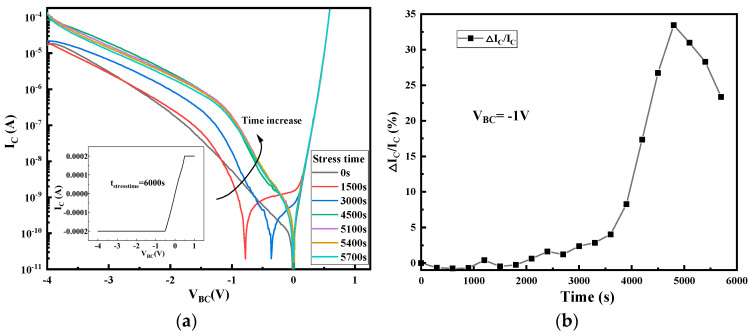
The degradation of B–C junction current during stress time: (**a**) the change in B–C junction current with stress time; (**b**) the degradation of B–C junction current at V_BC_ = −1 V.

**Figure 4:**
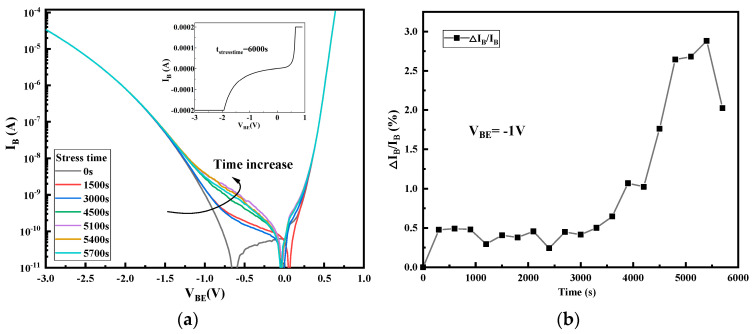
The degradation of B–E junction current during stress time: (**a**) the change in B–E junction current with stress time; (**b**) the degradation of B–E junction current at V_BE_ = −1 V.

Figure 3 and Figure 4 show the variation of B–E and B–C junction characteristics of the device with the increasing applicated stress time. We can see that the high reverse bias stress of the B–C junction makes both the B–C junction and B–E junction degenerate, but the degradation of the B–C junction is more obvious: the leakage current of the B–C junction changes with the stress time at the whole reverse bias conditions, while the B–E junction changes mainly under low reverse bias. Figure 3b and Figure 4b also show the amount of degradation of the reverse leakage current on the two junctions with a reverse bias of −1 V. Degradation quantity is defined as the relative increase (when compared to the non-stressed device) of current at different stress times at a given B–E junction bias voltage. As can be observed from the figure, the reverse bias leakage current of the B–E junction increases by 3.0 times, while the reverse bias leakage current of the B–C junction increases by 26.72 times after applying about 4800 s of stress, which indicates that the degradation of the B–C junction is faster than that of B–E junction. Moreover, it can also be seen from the figure that the degradation of the B–E junction also presents the same degradation trend as that of the B–C junction, which increases first and then decreases. Interior diagrams of Figure 3a and Figure 4a show the B–E and B–C junction characteristics after applying about 6000 s of stress; it can be found that the B–E junction has not been completely destroyed when the B–C junction has been broken down. This observation demonstrates a degradation process in which the high reverse bias of the B–C junction has an impact on the B–E junction even if there is no bias applied on the B–E junction during the stress, and the degeneration of the B–C junction is earlier than the B–E junction.

### 3.2. Effect of Stress on Device DC Characteristics

**Figure 5:**
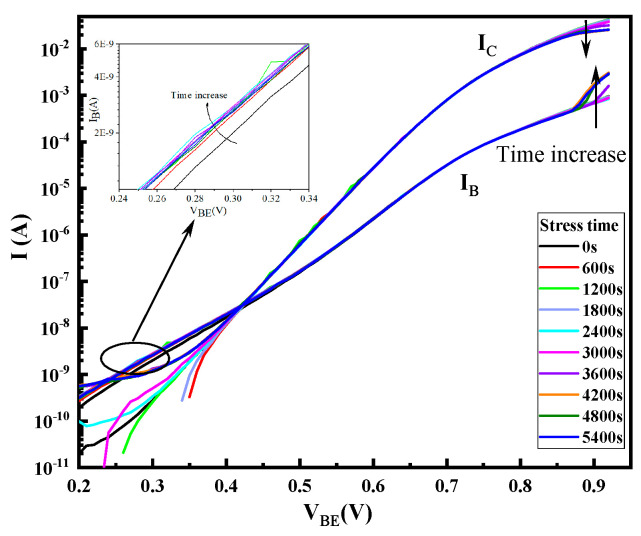
Changes of the Gummel plots with stress time.

**Figure 6:**
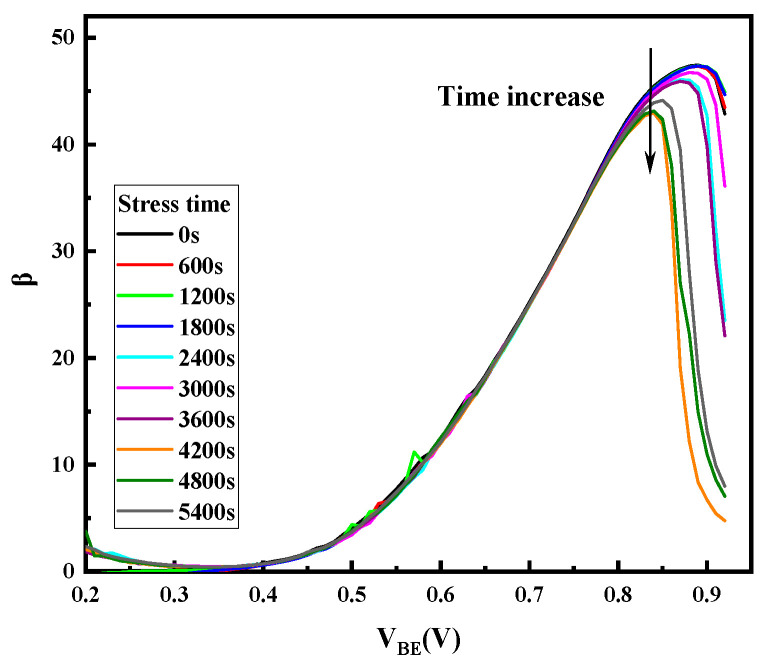
Changes of the current gain (β) with stress time.

**Figure 7:**
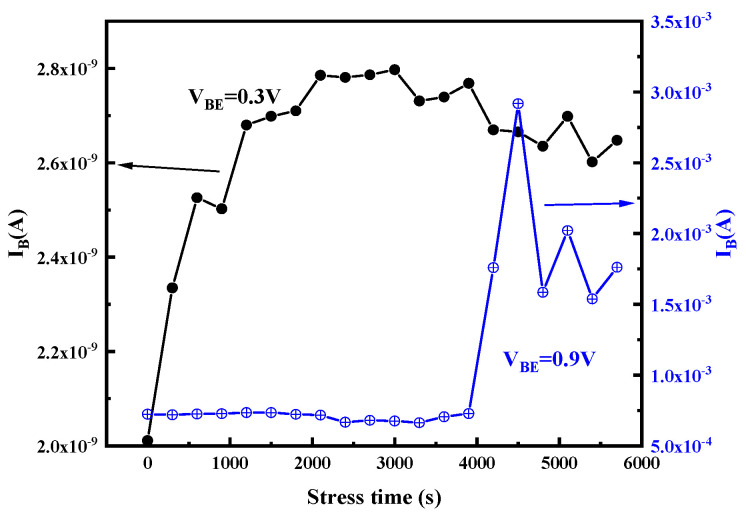
Plots of the changes of the base current with stress time at V_BE_ = 0.3 V and V_BE_ = 0.9 V.

**Figure 8:**
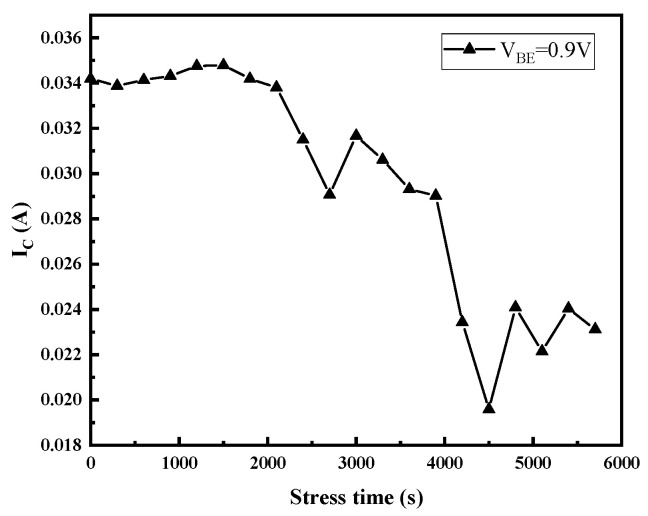
Plots of the changes of the collector current with stress time at V_BE_ = 0.9 V.

Figure 5 and Figure 6 display the gummel curves and variation of gain with increasing stress time, respectively. As can be seen from the figures, high reverse-bias stress mainly has an effect on base current I_B_, which mainly affects the base current in the lower voltage region (V_BE_ < 0.6 V) and higher voltage region (V_BE_ > 0.8 V). However, it only affects the collector current in the high-bias region (V_BE_ > 0.8 V). To analyze the degradation process of I_B_, the change in I_B_ with stress time in the low bias region (V_BE_ < 0.6 V) and the high bias region (V_BE_ > 0.8 V) of the forward gummel curve is extracted, which are shown in Figure 7. For the low-bias region (V_BE_ = 0.3 V), it can be observed that I_B_ presents two phases with the change in stress: during the stress, the base current increases rapidly and gradually reaches a maximum value (saturation value) within the first 2000 s and is then followed by a slight reduction as the stress time increases. However, for the high-bias region (V_BE_ = 0.9 V), I_B_ does not change during the first 4000 s or so and then rapidly increases. Figure 8 shows the evolution of I_c_ with stress time under stresses in the high voltage region (V_BE_ = 0.9 V), which indicates that I_c_ continuously decreases with the stress time. Therefore, the increase in base current and decrease in collector current lead to the decrease in current gain in the high bias region.

The base current of the device in the low-bias region is composed of various recombination currents. In InP/InGaAs HBT, the main bulk mechanism is the Auger recombination in the base [11]. Therefore, the rapid increase in base current in the first 2000 s might be mainly due to the increase in generation–recombination centers in the base. Subsequently, the generation–recombination centers (G-R centers) will reach the saturation value with the increasing stress time. The variation of I_B_ and I_c_ in the high-bias region is generally related to the degradation of series resistance. Therefore, the *S*-parameters of the device were also measured to extract the key parameter resistance of the device and establish the numerical device model to further analyze the degradation mechanism, which will be seen in the subsequent sections.

### 3.3. Effect of Stress on Device AC Small Signal Characteristics

The characteristic frequency *f_T_* and the maximum oscillation frequency *f*_max_ are the important parameters of RF microwave semiconductor devices, which are related to the resistance and capacitance inside the device [15,16]:

(1) $$f_{T}=\frac{1}{2\times \pi \times \tau_{ec}}$$

(2) $$f_{max}=\sqrt{\frac{f_{T}}{8\times \pi \times R_{bi}\times C_{bc}}}$$

with

(3) $$\tau_{ec}=\tau_{b}+\tau_{c}+\tau_{e}+\tau_{rc};\tau_{b}=\frac{W_{b}^{2}}{2\times (\frac{kT}{q}\times \mu_{nb})}+\frac{W_{b}}{V_{sat}}$$

(4) $$\tau_{c}=\frac{W_{c}}{2\times V_{sat}};\tau_{e}=R_{be}\times C_{be};\tau_{rc}=\left[R_{be}+R_{cc}+R_{ee}\right]\times C_{bc}$$

From Equation (1), we can see that *f_T_* is a function of total transit time (*τ_ec_*) required for the injected carriers from the emitter to reach the collector, while *τ_ec_* depends on intrinsic base resistance (*R_bi_*), collector access resistance (*R_c_*), emitter access resistance (*R_e_*), base–emitter capacitance (C_be_) and base–collector capacitance (*C_bc_*) according to Equations (3) and (4). The variation of fmax is determined by *f_T_*, *C_bc_* and intrinsic base resistance (*R_bi_*). Therefore, based on the S-parameters measured under different stresses, the key parameters such as junction capacitance and resistance are extracted to fully understand and analyze the high-field degradation mechanism of devices.

**Figure 9:**
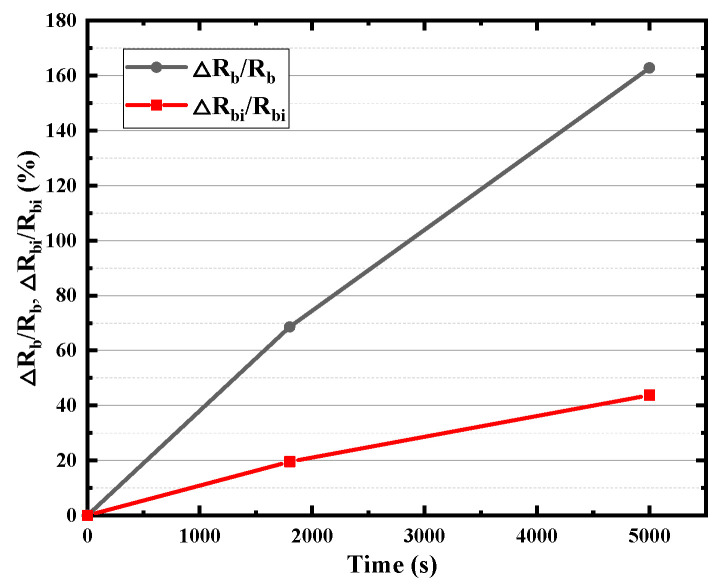
Degradation of *R_b_* and *R_bi_* with stress time.

**Figure 10:**
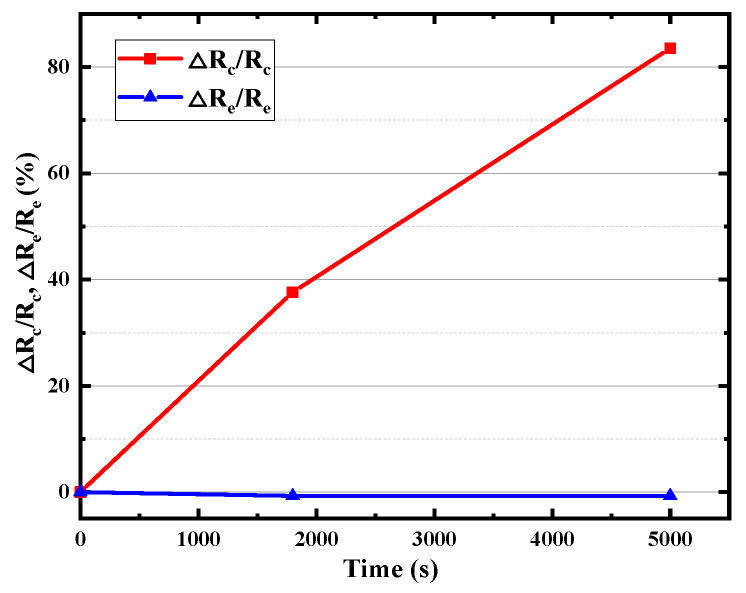
Degradation of *R_c_* and *R_e_* with stress time.

**Figure 11:**
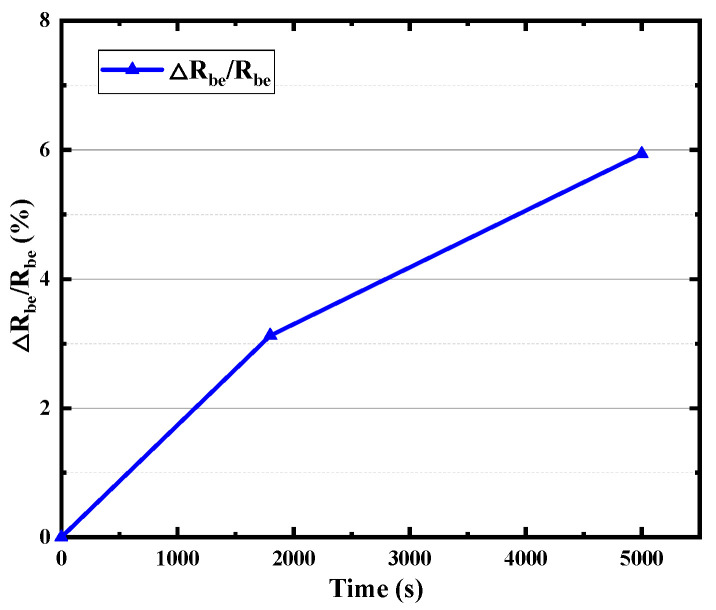
Degradation of *R_be_* with stress time.

**Figure 12:**
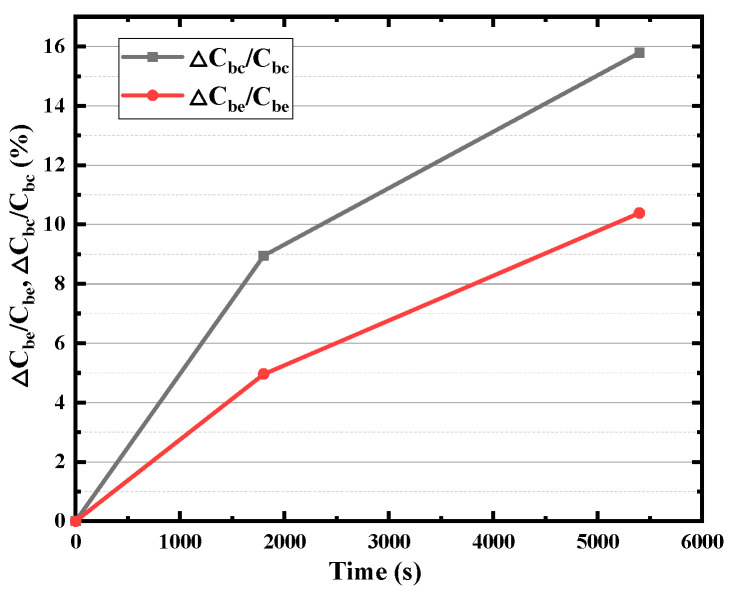
Degradation of *C_be_* and *C_bc_* with stress time.

The base region resistance, one of the most important electrical parameters of HBT, consists of inner (the intrinsic base resistance *R_bi_*) and outer parts (base resistance *R_b_*), which limits the charging rate of the input capacitance and thus limits the operation of the transistor at high frequencies. As shown in Figure 9, with the increase in stress time, both the *R_bi_* and *R_b_* increase with the stress time, while the degradation rate of *R_bi_* is smaller than that of *R_b_*. There is about an 80% rise in *R_bi_* but a more than 160% rise in *R_b_* after the 5000 s stress time. It can be found from Figure 10 that collector resistance *R_c_* also increases with stress time, while the emitter resistance *R_e_* hardly degrades. The B–E junction resistance *R_be_* has also been extracted and displayed in Figure 11, which increases with stress time but the degradation rate of *R_be_* is smaller than that of *R_bi_*. Device capacitance is mainly divided into two parts, B–C junction capacitance (*C_bc_*) and B–E junction capacitance (*C_be_*). As shown in the Figure 12, the extraction results show that both junction capacitances increase gradually with the increase in stress time, and the degradation of B–C junction capacitance is faster than that of B–E junction capacitance, which is probably due to the reverse high field of the B–C junction which is mainly applied to the B–C junction. The degradation of junction resistances and junction capacitances indicates once again that both B–E and B–C junctions are damaged by the reverse bias stress of the B–C junction, and the degradation of B–C junctions is even greater.

## 4. Degradation Mechanism Analysis with TCAD Device Simulation

### 4.1. Degradation Process: Device Degradation versus Stress Time

**Figure 13:**
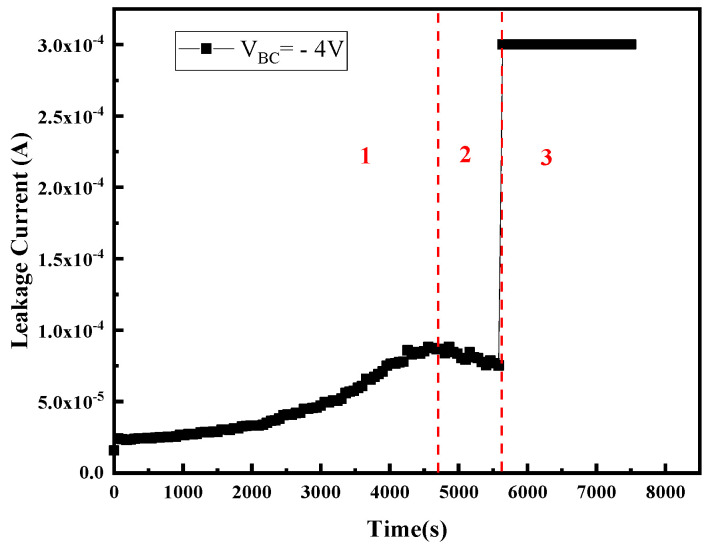
B–C leakage current versus time measured at V_BC_ = −2 V (stress voltage was V_BC_ = −4 V). Three degradation stages of the device were determined according to the plot of B–C leakage current versus time.

To analyze the degradation process of the device caused by the high reverse bias, the change curve of the reverse leakage current of the B–C junction with the stress time was monitored during the stress. One extreme value can be observed during the whole period of high-field stress. Figure 13 shows the typical time dependence of the B–C leakage current during the stress. It can be found that with the increase in stress time, the change in B–C junction leakage current exhibits three stages: The B–C junction leakage current increases with the increasing stress time and reaches a maximum within the first 4800 s and is then followed by a reduction of the leakage current. Subsequently, as the stress time increases, the B–C junction of the device is broken down. This feature suggests that three different degradation processes might be involved before the breakdown of the B–C junction of the device.

### 4.2. The Stress-Induced Degradation and Physical Model

**Figure 14:**
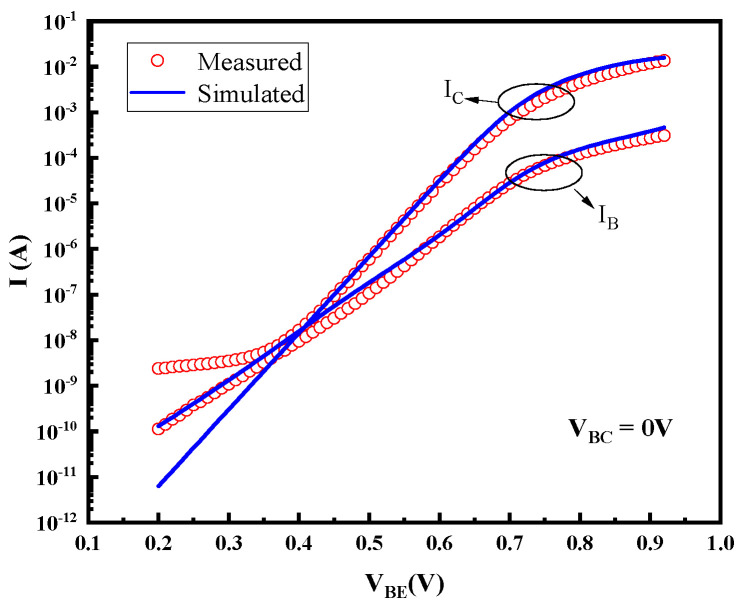
Comparison of measured and simulated gummel plots before stress.

**Figure 15:**
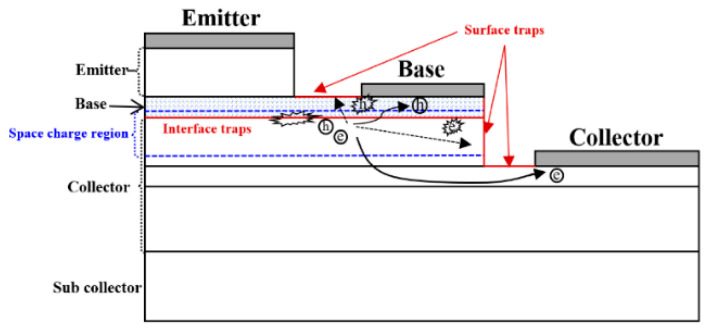
The physical degradation model of the device.

**Figure 16:**
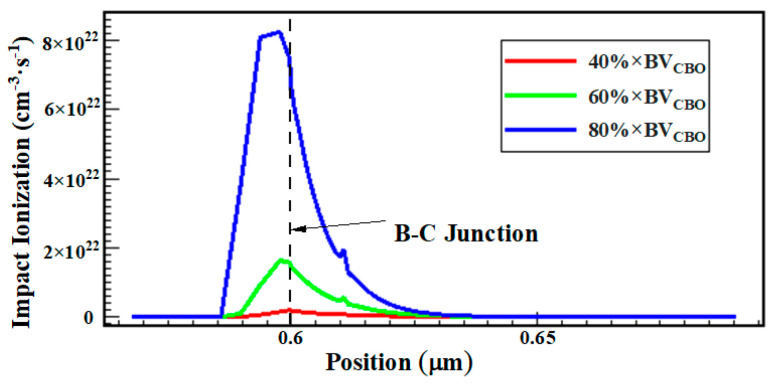
Relationship between impact ionization and reverse-biased stress.

**Figure 17:**
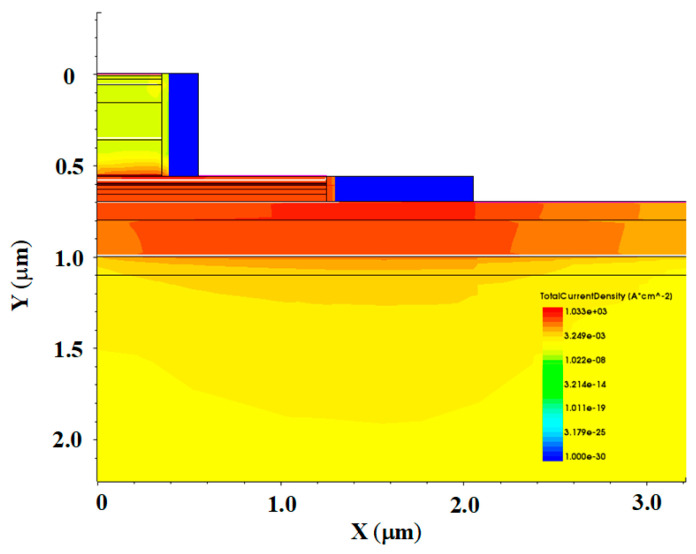
Total current density distribution under high-field stress.

In this paper, a numerical simulation model of the device (Figure 14) is established to analyze and interpret the high-field degradation mechanism of the device. Two-dimensional structure, hydrodynamic model, Shockley–Read–Hall (SRH) recombination, and Auger recombination models have been used [11,17,18]. To accurately simulate the base and the collector current, the surface traps with different energy levels located on the emitter sidewalls in the B–E junction and the B–C junction are introduced. Table 1 shows the location, the energy level in the bandgap, and the type of trap (acceptor or donor) used in the physical model. The trap location, type, and energy level were confirmed by RuizPalmero et al. [19]. Under stress, the majority of the carriers concentrated in the B–C junction, but also some of the carriers reached the B–E junction interface and passivation layers. Based on the device failure laws observed in experiments, we believe that the hot-carrier-induced device damage is the cause of the degradation mechanism, and a hot-carrier degradation model including induced device damage is proposed to explain the device degradation mechanism, as shown in Figure 15. The observed three failure stages are as follows:

(1) In the first stage, the B–C junction leakage current increases with the increasing stress time and reaches a maximum within the first 4800 s. In this stage, junction damage caused by hot carriers generated by the high field is the main cause of device degradation: the carriers will gain energy from the reversed high electric field region of the B–C junction to become the hot electrons and hot holes, and cause the impact ionization [13], resulting in damage to the collector junction. Figure 16 shows the distribution of impact ionization under the high reverse B–C bias stress, from which we can discover that the impact ionization occurs mainly in the space charge region of the B–C junction, and the impact ionization rate is even larger near the highly doped base region and B–C heterojunction. Moreover, the impact ionization rate is proportional to the electric field intensity, which increases with the increase in reverse bias, and the device will be broken down when a large enough electric field is applied. Therefore, the hot carriers increase the generation and recombination centers in the space charge zone of the base and B–C junction region, leading to the increase in the junction leakage current. As the generation and recombination centers caused by hot carriers have a maximum value (saturation value) [20], the leakage current reaches the maximum value when the stress is applied for 4800 s and does not increase.

(2) If the device degradation is dominated by device junction damage caused by hot carriers due to impact ionization, the leakage current of the B–C junction will continue to increase until breakdown occurs. However, there is clearly a stage II in which the B–C junction leakage decreases with the increasing stress time, likely due to the surface damage and contact resistance degradation caused by hot carriers. It can be observed from the internal carrier distribution of the device simulation model under the reverse high-field condition (Figure 17) that the carriers are mainly concentrated in the base region and collector region, which are mainly in the B–C junction, near the passivation layer and the contact electrode, and almost no carriers reach the emitter region. Therefore, in the process of reverse high field stress, although the majority of the hot carriers flow to the electrode, some of them may gain sufficient energy from the continuous high field stress of the B–C junction to inject to the B–E and B–C junction periphery and cause damage, or reach the surface regions of the base and collector, leading to the increase in the surface traps and the base resistance and collector resistance, or be trapped by the passivation layer to the distortion of surface potential distort the device surface potential (reduction of the junction field). Both the increase in resistance and distortion of the device’s surface potential will lead to a decrease in the reverse electric field applied at the B–C junction, leading to a decrease in the leakage current with the increase in stress time after reaching the maximum value.

(3) Stage III is the abrupt increase in current (breakdown). With the increase in the reverse high field stress time, the device undergoes a long period of defect generation and accumulation, and the amount of charge captured by the passivation layer also keeps increasing, resulting in the further expansion of the depletion zone. The increasing number of traps overlap with each other and gradually connect into a conductive channel, thus generating a conductive path between base and collector, leading to the breakdown of the B–C junction when the B–E junction has not been completely destroyed.

**Figure 18:**
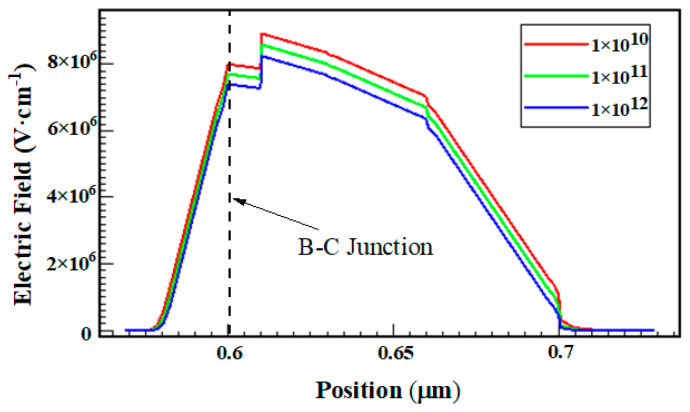
Electric field as a function of interface trap density.

**Figure 19:**
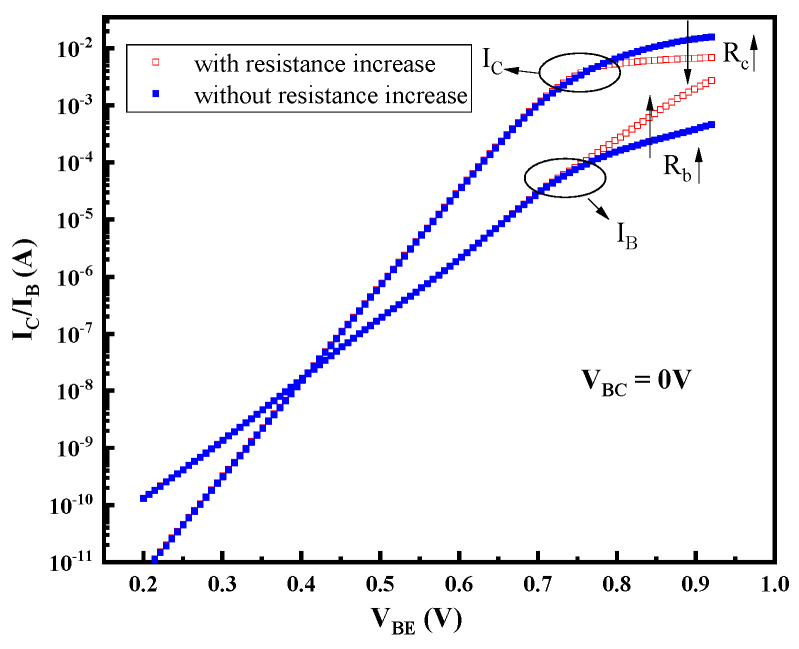
Comparison of simulated Gummel plots with and without resistance increase.

Based on this physical degradation model, the increase in junction capacitance can be mainly explained by two aspects: one is the increase in junction interface traps caused by the high field; the other one is the increase in the number of charges captured in the passivation layer and access resistances which leads to the decrease in the junction field imposed on the junction and narrowing the space charge zone. Figure 18 shows the relationship between the density of the interface acceptor traps and the distribution intensity of the electric field. The position where the electric field is zero is the boundary of the space charge region. It can be found that with the increase in the trap density, the maximum electric field decreases and the space charge region narrows, resulting in the capacitance increase. The degradation of I_B_ in the low-bias region can be explained by the increase in the generation of recombination centers, and the degeneration of R_bi_ mainly relates to the reduction of the carrier mobility in the base region caused by the increscent trap concentration. The degradation of gummel characteristics in the high-bias region is mainly related to the increased access resistances. The simulated gummel characteristics of devices with and without the inclusion of resistance degradation effects are shown in Figure 19, which confirms the degradation mechanism.

The lower degradation of the B–E junction is mainly due to energy relaxation. The hot carriers are generated in the high-reverse-bias B–C junction due to the impact ionization, the majority of the holes flow into the base electrode, and some of the holes with enough energy will cross the base layer to reach the B–E junction interface. However, since energy relaxation occurs, it reduces the energy of the holes when the holes cross the base layer, thus causing a delay in the degradation of the B–E junction [13]. That is why the high reverse bias of the B–C junction has an impact on the B–E junction even if there is no bias applied to the B–E junction during the stress.

## 5. Conclusions

In this paper, the impact of the high reverse-biased B–C junction stress on the reliability of InGaAs/InP DHBTs is investigated, and the three stages of device degradation are analyzed through the TCAD simulation. The results have indicated that the hot carriers are generated in the high-reverse-bias B–C junction due to the high electric field and impact ionization, which results in the damage of the B–C junction, the base and collector contact, and the collector–sidewall/passivation, leading to the increase in B–C junction leakage and the degeneration of the key device parameters. With the increase in the reverse high-field stress time, the increasing number of traps overlap with each other and gradually connect into a conductive channel, thus generating a conductive path between base and collector, leading to the breakdown of the B–C junction. The in-depth analysis of the high-field degradation mechanism is not only of great significance for designing InP DHBTs of high-power and high-reliability application requirements but also contributes to predicting and evaluating the reliability of existing circuits by adding failure mechanisms into the compact model.

## Figures and Tables

**Table 1:** Summarization of the model’s parameters in 2-D physical simulation.

Location	Type	E_T_-E_V_ (eV)
B–E junction	Acceptor	0.4
B–C junction	Acceptor	0.4
Emitter sidewall	Donor	0.83 1.3

## Data Availability

Data are contained within the article.
